# Toxicity classification of e-cigarette flavouring compounds based on European Union regulation: analysis of findings from a recent study

**DOI:** 10.1186/s12954-019-0318-2

**Published:** 2019-07-25

**Authors:** Konstantinos Farsalinos, George Lagoumintzis

**Affiliations:** 1 Department of Cardiology, Onassis Cardiac Surgery Center, Syggrou 356, 17674 Kallithea, Greece; 2 Laboratory of Molecular Biology and Immunology, Department of Pharmacy, University of Patras, 26500 Rio, Greece; 3 National School of Public Health, Athens, Greece

**Keywords:** Electronic cigarettes, Liquids, Flavourings, Toxicity, European Union

## Abstract

**Introduction:**

A recent study raised concerns about e-cigarette liquids toxicity by reporting the presence of 14 flavouring chemicals with toxicity classification. However, the relevant toxicity classification was not estimated according to the measured concentrations. The purpose of this study was to calculate the toxicity classification for different health hazards for all the flavouring chemicals at the maximum concentrations reported.

**Methods:**

The analysis was based on the European Union Classification Labelling and Packaging regulation. The concentration of each flavouring chemical was compared with the minimum concentration needed to classify it as toxic. Additionally, toxicity classification was examined for a theoretical e-cigarette liquid containing all flavouring chemicals at the maximum concentrations reported.

**Results:**

There was at least one toxicity classification for all the flavouring chemicals, with the most prevalent classifications related to skin, oral, eye and respiratory toxicities. One chemical (methyl cyclopentenolone) was found at a maximum concentration 150.7% higher than that needed to be classified as toxic. For the rest, the maximum reported concentrations were 71.6 to > 99.9% lower than toxicity concentrations. A liquid containing all flavouring compounds at the maximum concentrations would be classified as toxic for one category only due to the presence of methyl cyclopentenolone; a liquid without methyl cyclopentenolone would have 66.7 to > 99.9% lower concentrations of flavourings than those needed to be classified as toxic.

**Conclusions:**

The vast majority of flavouring compounds in e-cigarette liquids as reported in a recent study were present at levels far lower than needed to classify them as toxic. Since exceptions exist, regulatory monitoring of liquid composition is warranted.

## Background

Smoking is the foremost risk factor for many human diseases and promotes the initiation as well as the progression of potential lethal illnesses such as chronic obstructive pulmonary disease, cardiovascular disease and lung cancer [1]. While pharmacotherapies for smoking cessation have been developed for several years, their popularity and success rate is limited [2–4]. As a result, tobacco harm reduction products have been developed, with electronic cigarettes (e-cigarettes) being the most popular and widely used globally.

E-cigarette devices are comprised of a battery with integrated electronics and an atomizer that includes a wick, a heating element and a liquid storage space. Typically, e-cigarette liquids contain water, nicotine, vegetable glycerin (VG), propylene glycerol (PG) and a mix of flavouring additives, in variable concentrations, in order to achieve the desired taste while vaping [5]. Today, there is a vast choice of e-cigarette liquids, with a wide range of flavourings and nicotine levels. Besides the traditional tobacco-like flavours, consumers can choose among a variety of flavours consisting of fruits, sweets, drinks and beverages, and many more. Flavouring additives are used because e-cigarettes are almost flavourless without them. Surveys have shown that the vast majority of e-cigarette users use flavoured liquids, change flavours frequently, and report that flavour variability is important in their effort to quit and stay off cigarettes [6, 7].

Almost all flavouring chemicals are substances Generally Recognized As Safe (GRAS) and approved for human consumption through the oral route. While this does not substantiate safety for inhalation, food-approved flavourings are the only source for flavouring compounds used in e-cigarettes. A recent study by Vardavas et al. raised concerns about the presence of flavouring additives in e-cigarette liquids [8]. The study reported the analysis of 122 samples identifying several chemical compounds that are classified according to health hazards, including classification as respiratory irritants. This raised the possibility for toxicity and the legal requirement to include appropriate labelling for toxicity based on established European Union (EU) regulations. The authors noted that the liquids tested did not comply with the current EU regulations on e-cigarettes (Tobacco Products Directive) which dictates that “with the exception of nicotine, only ingredients that do not pose a risk to human health in heated or unheated form should be used in the liquid” [9]. There are established methods of identifying and classifying the toxicity of chemicals and mixtures based on the European Chemicals Agency Classification Labelling and Packaging (CLP) regulation, which are relevant to all products available for human consumption, including e-cigarette liquids [10, 11]. The toxicity characterization depends on the toxicity classification of the compounds and the concentration of the chemical in the mixture, in compliance with a basic toxicological principle that the amount of exposure determines the toxicity [12]. Although the flavouring chemicals were identified and quantified, the study did not calculate the potential toxicity and relevant toxicity classification based on the concentrations of the chemicals. Therefore, the purpose of this study was to examine the toxicity classification for different health hazards for all the chemicals at the maximum concentrations, as reported by Vardavas et al., and to determine if there is a legal requirement to include warning labels to the products according to flavouring levels, as determined by established regulation.

## Methods

### Toxicity classification methodology

Toxicity classification for all chemicals reported by Vardavas et al. was sought in the classification and labelling information database of the European Chemicals Agency [13]. The EU provides clear guidance on the estimation of toxicity of chemicals and mixtures through the CLP regulation [10, 11]. Each chemical is classified according to different hazards. There are mainly 3 types of hazard classes: physical hazards, health hazards and environmental hazards [14]. Based on the type of hazard and toxicity classification, specific hazard statements are required each of which has a specific code [15].

Health hazards include acute toxicity for oral, dermal and inhalation exposure, with chemicals being allocated to one of four toxicity categories (Category 1 to 4) according to specific numeric criteria. For these health hazards, acute toxicity values are expressed as approximate LD50 values or as acute toxicity estimates (ATE) from experimental data [10]. The classification categories are defined according to dose cut-off values of chemicals (in mg/kg body weight) causing toxicity in animals, with higher dose needed to cause toxicity corresponding to lower toxicity classification. E-cigarette liquids are mixtures, with flavouring chemicals diluted in non-toxic solvents (PG and VG). Therefore, the method of classification of mixtures for toxicity was used to identify the toxicity classification of each chemical at the concentrations reported by Vardavas et al. Since test data on the mixture itself or similar mixtures are not available, the classification was based on calculation thresholds. Two types of analyses were performed. In one, each compound reported in the study by Vardavas et al. [8] was assumed to be the only component of the mixture dissolved in non-toxic solvents (PG and VG) at the maximum levels reported by Vardavas et al. A limitation of this method is that electronic cigarette liquids contain more than one flavouring chemical that could have a toxicity classification. Unfortunately, the previous study did not provide information on the composition of each of the liquids tested. Thus, and to address this limitation, we calculated the toxicity estimate for a hypothetical final solution (e-cigarette liquid) containing all the flavouring chemicals at the maximum concentration reported by Vardavas et al. This is performed using the additivity formula (10), which involves adding the Acute Toxicity Estimate (ATE) of each ingredient for each hazard classification, in this case of all compounds at the maximum concentrations reported by Vardavas et al. While it is unlikely that an e-cigarette liquid would contain all these flavouring chemicals at the maximum reported concentrations, it provides an estimate of the worst-possible case scenario based on the study findings. Thus, to estimate the hazard classification for mixtures, we used the following formula:

$$ \frac{100}{ATEmix}=\frac{\sum \limits_n Ci}{ATEi} $$

where ATEmix is the acute toxicity estimate of the mixture containing a specific concentration of the chemical, *n* is the number of ingredients (one ingredient for the analysis of each compound separately and sum of all ingredients in the analysis of a liquid containing all ingredients in maximum concentrations), Ci is the concentration of the chemical *i* in the mixture, and ATEi is the converted acute toxicity point estimate of chemical *i*. Since toxicity classification to different categories is based on a range of acute toxicity estimates, we used the converted acute toxicity point estimates as recommended by the CLP regulation in the calculations [10]. It should be clarified that lower ATEi represents lower exposure dose needed to cause toxicity and thus higher toxicity classification (i.e. more toxic). Similarly, lower ATEmix represents lower exposure dose of the mixture needed to cause toxicity and thus higher toxicity.

For other health and for environmental hazards, such as skin corrosion and irritation, eye irritation, respiratory irritation and toxicity to aquatic life, percent concentrations of the chemical are used to determine the different toxicity categories (Table 2). For these hazards, the maximum concentrations reported by Vardavas et al. were used. Separate analyses were performed considering that each chemical represents a unique mixture with the chemical present at the maximum concentration and assuming that an e-cigarette liquid contains all ingredients at the maximum concentrations reported. In the latter case, the concentrations of each compound having a specific toxicity classification were added in order to calculate the toxicity classification for the final mixture.

## Results

Table 1 displays the chemicals (*n* = 14) and the maximum concentrations reported by Vardavas et al., as well as their toxicity classification according to the EU CLP [15–28]. All chemicals were approved to be used as food flavourings, and their respective Flavor Extract Manufacturers Association (FEMA) GRAS numbers are also displayed in Table 1. There was at least one toxicity classification for all the flavouring chemicals. The most prevalent health hazards were related to skin (5 chemicals classified as skin irritants and 4 classified as causing allergic skin reactions), oral (6 chemicals), and eye (5 chemicals) and respiratory (3 for respiratory irritation and 2 for allergy, asthma symptoms or breathing difficulties) toxicity. While 3 chemicals were classified as flammable at ≤ 60 °C in liquid and vapour form, e-cigarette liquids are obviously not flammable even when being heated to the temperatures of evaporation during use. Thus, no further analysis was performed for this hazard category. It should be noted that ethyl hexanoate was only classified as flammable, so it was excluded from further analysis.

**Table 1:** Hazard and hazard category classifications of chemicals reported by Vardavas et al. and their corresponding FEMA numbers

Substance	FEMA GRAS No	Hazard, hazard category and hazard phrase according to CLP	Reference
Menthol	2265	H315 Causes skin irritation (Category 2)	[16]
		H319 Causes serious eye irritation (Category 2)	
Ethyl maltol	3487	H302 Harmful if swallowed (Category 4)	[15]
Linalool	2635	H315 Causes skin irritation (Category 2)	[17]
		H317 May cause an allergic skin reaction. (Category 1)	
		H319 Causes serious eye irritation (Category 2)	
Methyl cyclopentenolone	2700	H302 Harmful if swallowed (Category 4)	[18]
		H319 Causes serious eye irritation (Category 2)	
		H334 May cause allergy or asthma symptoms or breathing difficulties if inhaled (Category 1)	
		H335 May cause respiratory irritation (Category 3)	
*β*-Damascone	3243	H317 May cause an allergic skin reaction. (Category 1)	[19]
		H411 Toxic to aquatic life with long-lasting effects (Category 2)	
Ethyl vanillin	2464	H302 Harmful if swallowed (Category 4)	[20]
		H315 Causes skin irritation (Category 2)	
		H319 Causes serious eye irritation (Category 2)	
		H335 May cause respiratory irritation (Category 3)	
		H412 Harmful to aquatic life with long-lasting effects (Category 3)	
*β*-Ionone	2595	H400 Very toxic to aquatic life (Category 1)	[21]
		H411 Toxic to aquatic life with long-lasting effects (Category 2)	
Acetyl pyrazine	3126	H315 Causes skin irritation (Category 2)	[22]
		H319 Causes serious eye irritation (Category 2)	
		H335 May cause respiratory irritation (Category 3)	
*α*-Ionone	2594	H334 May cause allergy or asthma symptoms or breathing difficulties if inhaled (Category 1)	[23]
		H412 Harmful to aquatic life with long-lasting effects (Category 3)	
Ethyl hexanoate	2439	H226 Flammable liquid and vapour (Category 3)	[24]
2,5 dimethylpyrazine	3271	H226 Flammable liquid and vapour (Category 3)	[25]
		H302 Harmful if swallowed (Category 4)	
*α*-Damascone	4088	H302 Harmful if swallowed (Category 4)	[26]
		H317 May cause an allergic skin reaction (Category 1)	
		H411 Toxic to aquatic life with long-lasting effects (Category 2)	
3,4 Dimethoxy-benzaldehyde	3109	H302 Harmful if swallowed (Category 4)	[27]
Limonene	2633	H226 Flammable liquid and vapour (Category 3)	[28]
		H304 May be fatal if swallowed and enters airways (Category 1)	
		H315 Causes skin irritation (Category 2)	
		H317 May cause an allergic skin reaction (Category 1)	
		H400 Very toxic to aquatic life (Category 1)	
		H410 Very toxic to aquatic life with long-lasting effect (Category 1)	

Table 2 presents the toxicity classification calculations for each compound separately using the maximum concentrations reported by Vardavas et al. [8]. Only methyl cyclopentenolone was found at a maximum concentration that would result in toxicity classification for one hazard (H 334 Category 1, may cause allergy or asthma symptoms or breathing difficulties if inhaled). All other compounds were found at maximum concentrations that were by far lower than the concentrations needed to classify them as toxic. The difference between the minimum concentrations needed to classify a solution as toxic and the maximum concentrations reported by Vardavas et al. ranged from approximately 72% (for ethyl maltol) to > 99.9% (250.000-fold lower concentration for limonene).

**Table 2:** Toxicity classification calculations for each flavouring compound using the maximum concentrations reported by Vardavas et al.

Substance	Hazard category	Reported max concentration (% *w*/*w*)*	Calculated ATEmix	ATEmix limit or min concentration for toxicity classification	% difference**
Menthol	Η315 Category 2	0.4991		10	− 95.0%
	Η319 Category 2	0.4991		10	− 95.0%
Ethyl maltol	Η302 Category 4	7.096	7046	≤ 2000	− 71.6%
Linalool	Η315 Category 2	0.263		10	− 97.4%
	Η317 Category 1	0.263		1	− 73.7%
	Η319 Category 2	0.263		10	− 97.4%
Methyl cyclopentenolone	Η302 Category 4	2.5067	19,946	≤ 2000	− 90.0%
	Η319 Category 2	2.5067		10	− 74.9%
	*Η334 Category 1*	*2.5067*		*1*	*150.7%*
	Η335 Category 3	2.5067		20	− 87.5%
*β*-Damascone	Η317 Category 1	0.0742		1	− 92.6%
	Η411 Category 2	0.0742		25	− 99.7%
Ethyl vanillin	Η302 Category 4	0.9135	54,734	≤ 2000	− 96.3%
	Η315 Category 2	0.9135		10	− 90.9%
	Η319 Category 2	0.9135		10	− 90.9%
	Η335 Category 3	0.9135		20	− 95.4%
	Η412 Category 3	0.9135		25	− 96.3%
*β*-Ionone	Η400 Category 1	0.0076		25	> − 99.9%
	Η411 Category 2	0.0076		25	> − 99.9%
Acetyl pyrazine	Η315 Category 2	0.1067		10	− 98.9%
	Η319 Category 2	0.1067		10	− 98.9%
	Η335 Category 3	0.1067		20	− 99.5%
*α*-Ionone	Η334 Category 1	0.012		1	− 98.8%
	Η412 Category 3	0.012		25	> − 99.9%
2,5 Dimethylpyrazine	Η302 Category 4	0.0197	2,538,071	≤ 2000	− 99.9%
*α*-Damascone	Η302 Category 4	0.0078	6,410,256	≤ 2000	> − 99.9%
	Η317 Category 1	0.0078		1	− 99.2%
	Η411 Category 2	0.0078		25	> − 99.9%
3,4 Dimethoxy-benzaldehyde	Η302 Category 4	0.0359	1,392,757	≤ 2000	− 99.9%
Limonene	Η304 Category 1	0.0001		10	> − 99.9%
	Η315 Category 2	0.0001		10	> − 99.9%
	Η317 Category 1	0.0001		1	> − 99.9%
	Η400 Category 1	0.0001		25	> − 99.9%
	Η410 Category 1	0.0001		25	> − 99.9%

*Maximum concentrations reported by Vardavas et al

**Percent difference between the maximum concentration reported by Vardavas et al. and the concentration needed to be classified as toxic

Italics represents the compound and hazard exceeding the limits for toxicity classification

Table 3 presents the toxicity classification for the worst case scenario of an e-cigarette liquid containing all the flavouring compounds reported by Vardavas et al., at the maximum concentrations found. The final liquid would need to be classified as H334 Category 1 (may cause allergy or asthma symptoms or breathing difficulties if inhaled). To determine whether the classification was solely based on the maximum concentration of methyl cyclopentenolone, we performed another calculation adding the concentrations and ATEs of all flavouring compounds besides methyl cyclopentenolone. The findings are presented in Table 4. The final liquid would not be classified as toxic for any health or environmental hazard.

**Table 3:** Toxicity classification of a mixture containing all flavouring chemicals at the maximum concentrations reported by Vardavas et al.

Classification hazard	ATEmix or total concentration (%) of all compounds with the same hazard classification	ATEmix limit or minimum concentration for classification of toxicity	% difference^1^
Η302 Category 4	4726	≤ 2000	− 57.7%
Η304 Category 1	0.0001	10	> − 99.9%
Η315 Category 2	1.7824	10	− 82.2%
Η317 Category 1	0.3451	1	− 65.5%
Η319 Category 2	4.289	10	− 57.1%
*Η334 Category 1*	*2.5187*	*1*	*151.9%*
Η335 Category 3	3.5269	10	− 64.7%
Η400 Category 1	0.0077	25	> − 99.9%
Η410 Category 1	0.0001	25	> − 99.9%
Η411 Category 2	0.0896	25	− 99.6%
Η412 Category 3	0.9255	25	− 96.3%

^1^Percent difference between the maximum concentration reported by Vardavas et al. and the concentration needed to be classified as toxic

Italics represents the compound and hazard exceeding the limits for toxicity classification

**Table 4:** Toxicity classification of a mixture containing all flavouring chemicals at the maximum concentrations reported by Vardavas et al. except methyl cyclopentenolone

Classification hazard	ATEmix or total concentration (%) of all compounds with the same hazard classification	Maximum ATEmix or minimum concentration for classification of toxicity	% difference^1^
Η302 Category 4	6193	2000	− 67.7%
Η304 Category 1	0.0001	10	> − 99.9%
Η315 Category 2	1.7824	10	− 82.2%
Η317 Category 1	0.3451	1	− 65.5%
Η319 Category 2	1.7823	10	− 82.2%
Η334 Category 1	0.012	1	− 98.8%
Η335 Category 3	1.0202	10	− 89.8%
Η400 Category 1	0.0077	25	> − 99.9%
Η410 Category 1	0.0001	25	> − 99.9%
Η411 Category 2	0.0896	25	− 99.6%
Η412 Category 3	0.9255	25	− 96.3%

^1^Percent difference between the maximum concentration reported by Vardavas et al. and the concentration needed to be classified as toxic

## Discussion

The study presented a risk assessment analysis of previously reported findings of flavouring chemicals in e-cigarette liquids. The analysis was based on established methods of classifying toxicity determined by relevant EU regulations and according to official toxicity classifications. The study found that only one flavouring compound (methyl cyclopentenolone) was present at a maximum concentration that would result in toxicity classification and the need to introduce specific warning labels based on established regulations. For all other compounds, the maximum concentrations were far below the levels needed to result in any toxicity classification. Even in the unlikely scenario that an e-cigarette liquid would contain all the flavouring compounds at the maximum reported concentrations, only methyl cyclopentenolone was present in sufficient concentration to classify the mixture as toxic.

It is not uncommon for many chemicals used for human consumption to be classified as toxic. Characteristically, ethyl vanillin, a very common flavouring used in food products, has a toxicity classification for oral intake (harmful if swallowed), a toxicity relevant to the intended route of intake (ingestion). Still, it is widely used in the food industry, with the annual production estimated at 44 tonnes in Europe and 330 tonnes in the USA [29]. This is because any toxicity classification is not based on the presence of a chemical alone but on the amount used in the final product and how this compares with concentrations associated with toxicity. Similarly, it is not unexpected that e-cigarette liquids contain chemicals that are classified for toxicity since flavourings used in these products are derived from the food industry. While the EU dictates that no chemical posing health risk (besides nicotine) should be used in e-cigarette liquids, it is expected that the same principles are applied to e-cigarettes as to all other consumer products (e.g., food). The previously published analysis by Vardavas et al. did not calculate the potential toxicity and relevant toxicity classification based on the concentrations of the chemicals. Their methodology would not be appropriate for other consumer products, and the current regulatory framework for chemical compounds used in consumer products makes no qualitative or quantitative distinction in the toxicity evaluation between e-cigarette liquids and other products, including food products.

Our analysis identified that the maximum concentration found for methyl cyclopentenolone was high enough to be classified as toxic. Previous studies have also identified that some e-cigarette liquids contain flavouring chemicals at levels exceeding the Maximized Survey-Derived Intake (MSDI) levels set by the EU or the USA or recommended levels in food products [30, 31]. These findings indicate that some manufacturers do not conform to proper manufacturing standards and introduce high levels of certain flavouring chemicals in e-cigarette liquids. While that was observed with only one chemical at the maximum concentration herein, it is important to implement proper regulatory and monitoring frameworks so that product quality and safety is ensured.

Limitations of the study include the lack of information about the composition of e-cigarette liquids so that a toxicological assessment of the final product rather than each compound separately would be performed. To overcome this, we analyzed a hypothetical, highly unlikely, scenario that a liquid would contain all chemicals at the maximum concentration reported by Vardavas et al. Even in this case, only methyl cyclopentenolone was associated with a toxicity classification. Additionally, Vardavas et al. reported finding 246 different flavours and additives in the 122 samples tested [8]. A recent study by the same group [32] presented more chemicals found in these e-cigarette liquids, but did not provide information on the concentrations found; thus, it was impossible to calculate the toxicity classifications for all these compounds. Finally, despite using the current regulatory framework for chemicals safety (CLP), this does not imply that e-cigarettes are harmless. Studies specifically addressing the route of exposure from e-cigarette use and the process of aerosol production (liquid heating and potential presence of thermal degradation products) are needed to examine the safety/risk profile of e-cigarettes. While the products are sold in liquid form, they are evaporated and the generated aerosol in subsequently used by the consumer. Additionally, CLP regulation is mainly focused on acute toxicity while e-cigarettes are used chronically. Thus, the study cannot evaluate the safety/risk profile of these products from chronic intended use. However, it provides an assessment of the products’ compliance with established chemicals safety regulations in the EU, of the legal requirements to include warning labelling based on established toxicity regulation and of the compliance to the TPD regulation which dictates that no compound with a toxicity classification (besides nicotine) should be added to e-cigarette liquids.

## Conclusions

In conclusion, a risk assessment analysis based on the EU CLP regulatory framework identified that only one of the reported flavouring chemicals found in e-cigarette liquids was present at levels sufficiently high to classify it as toxic based on the EU regulatory framework. For the rest, the concentrations reported were by far lower than those needed to classify them as toxic. Even if a liquid contained all the chemical compounds at the maximum reported concentration, still any toxicity classification would be associated with the use of only one compound at the maximum concentration. It is important for a proper regulatory framework to continuously monitor the composition and quality of e-cigarette products available in the market and ensure that appropriate standards are used. Such toxicological surveillance of e-cigarette liquids can be valuable in identifying, removing or adequately diluting potentially harmful compounds as part of standard regulatory practice. The relative simplicity of the chemistry of e-liquids and e-cigarette aerosols makes this method practically feasible, whereas it is totally implausible for combustible tobacco products.

## Data Availability

All data generated or analyzed during this study are included in this published article.

## Abbreviations

ATE: Acute toxicity estimate
CLP: Classification Labelling and Packaging
E-cigarettes: Electronic cigarettes
EU: European Union
FEMA: Flavor Extract Manufacturers Association
GRAS: Generally Recognized As Safe
LD50: Lethal dose 50
MSDI: Maximized Survey-Derived Intake
PG: Propylene glycerol
USA: United States of America
VG: Vegetable glycerin
WHO: World Health Organization
